# Effects of the Manual Therapy Approach of Segments C0-1 and C2-3 in the Flexion-Rotation Test in Patients with Chronic Neck Pain: A Randomized Controlled Trial

**DOI:** 10.3390/ijerph18020753

**Published:** 2021-01-17

**Authors:** Jacobo Rodríguez-Sanz, Miguel Malo-Urriés, María Orosia Lucha-López, Albert Pérez-Bellmunt, Andoni Carrasco-Uribarren, Pablo Fanlo-Mazas, Jaime Corral-de-Toro, César Hidalgo-García

**Affiliations:** 1 Faculty of Medicine and Health Sciences, Universitat Internacional de Catalunya, C/Josep Trueta s/n, Sant Cugat del Vallés, 08195 Barcelona, Spain; aperez@uic.es (A.P.-B.); acarrasco@uic.es (A.C.-U.); 2 Departamento de Fisiatría y Enfermería, Unidad de Investigación en Fisioterapia, Facultad de Ciencias de la Salud, Universidad de Zaragoza, C/Domingo Miral, s/n, 50009 Zaragoza, Spain; malom@unizar.es (M.M.-U.); orolucha@unizar.es (M.O.L.-L.); pfanlo@unizar.es (P.F.-M.); jaimecorral.fisio@gmail.com (J.C.-d.-T.); hidalgo@unizar.es (C.H.-G.)

**Keywords:** manual therapy, neck pain, exercise, restriction

## Abstract

*Background*: Flexion-rotation test predominantly measures rotation in C1-2 segment. Restriction in flexion-rotation may be due to direct limitation in C1-2, but also to a premature tightening of the alar ligament as a result of lack of movement in C0-1 or C2-3. The aim of this study was to compare the effect of a 20-min single cervical exercise session, with or without manual therapy of C0-1 and C2-3 segment in flexion-rotation test, in patients with chronic neck pain and positive flexion-rotation test. *Methods*: Randomized controlled clinical trial in 48 subjects (24 manual therapy+exercise/24 exercise). Range of motion and pain during flexion-rotation test, neck pain intensity and active cervical range of motion were measured before and after the intervention. *Results*: Significant differences were found in favour of the manual therapy group in the flexion-rotation test: right (*p* < 0.001) and left rotation (*p* < 0.001); pain during the flexion-rotation test: right (*p* < 0.001) and left rotation (*p* < 0.001); neck pain intensity: (*p* < 0.001); cervical flexion (*p* < 0.038), extension (*p* < 0.010), right side-bending (*p* < 0.035), left side-bending (*p* < 0.002), right rotation (*p* < 0.001), and left rotation (*p* < 0.006). *Conclusions*: Addition of one C0-C1 and C2-C3 manual therapy session to cervical exercise can immediately improve flexion-rotation test and cervical range of motion and reduce pain intensity.

## 1. Introduction

Mobility restrictions of the upper cervical spine have been associated with neck pain and headaches [1,2]. Most rotational movement of the upper cervical spine occurs at the C1-2 segment [3]. The flexion-rotation test is the most used test to assess the range of movement (ROM) in the transverse plane of the upper cervical spine, and it is a valid and reliable test [4,5,6,7]. This test is an easily applied method of manual examination that localizes the presence of joint dysfunction at C1-2 level [8]. During the flexion-rotation test, the head and neck are placed in maximal flexion, and the head and neck are turned left and right [3]. The usual ROM is 45° for each side [9]. The flexion-rotation test is positive if the range is less than 33° to one side or if there is a difference of 10° between sides [8,9].

Although it is considered a test to assess the rotation of the C1-2 segment, the presence of painful joints in the lower cervical spine reduced the available range in the flexion-rotation test to 37.5° [10]. This indicates that lower cervical spine pain may reduce the ROM recorded during the flexion-rotation test [11].

Takasaki et al. [5] recruited 19 subjects and examined the measurement reliability of segmental upper cervical movements using magnetic resonance imaging. They investigated the content validity of the flexion-rotation test comparing cervical rotation ROM of each segment from a neutral and a maximally flexed position of the neck. These researchers found 1.7° (1.9%) of movement in C0-1, 65° (73.5%) in C1-2, 2.6° (3%) in C2-3, and 19.1° (21.6%) between C3 and C7 during the flexion-rotation test. Smith et al. [12] determined that age, gender and lifestyle factors did not influence the flexion-rotation test. In turn, Hall et al. [13] found an inverse relation between severity of symptoms and flexion-rotation test ROM. Chronic neck pain alters the recruitment of the neck muscles [14], which can lead to decreased range of movement [15]. Mechanisms are not clear but it has been related to a reduction of the excitability of both cortical and spinal motor neurones [16].

Some studies have investigated the effectiveness of treating upper cervical spine restriction in the flexion-rotation test with joint mobilization and manipulation. These studies used joint manipulation and mobilization that was applied directly to the C1-2 segments at the end of the cervical rotation ROM [2,17,18]. This treatment is not without controversy within the literature. This is due to the unique ROM characteristics and vertebral artery angulation at the C1-2 motion segment. The portion of the vertebral artery between C1-2 is considered by some to be the most vulnerable site for injury [19], especially during rotational manipulation [20] and in addition to slow and repeated mobilization [21]. There is controversy over whether adverse events can be attributed to the upper cervical rotation. Therefore, the International Federation of Orthopedic Manual Physical Therapists (IFOMPT) recommended that end-range rotation and extension positions should be avoided during cervical spine mobilization and manipulation [22]. Alternative therapeutic strategies proposed in the literature to avoid the direct mobilization of C1-C2 are: thoracic spine thrust manipulation, exercise and patient education [23,24,25] and the mobilization of C0-C1 [1,3] and C2-C3 [6].

Two previous studies have shown that the mobilization of the C0-1 segment produced an improvement in flexion-rotation test [1,3]. Hidalgo-García et al. [26,27] explained that the upper cervical spine is a functional element from C0 to C3. The restriction of ROM in the flexion-rotation test, in some patients, could be explained by a premature tightening of the alar ligament as a result of lack of movement in the C0-C1 [26] and C2-3 segments [27].

However, there are no studies that have analysed the immediate effect of the manual therapy treatment of the C0-1 and C2-3 segment to improve the mobility in a restricted flexion-rotation test, without treating the C1-2 segment.

Cleland et al. suggest in different articles [23,24,25] that during the initial treatment sessions, there is a substantial probability of improved patient cervical ROM with indirect treatment, for example, when thoracic manipulation is coupled with cervical active ROM exercises [22]. This approach allows the therapist to observe the patient’s response to treatment, and theoretically minimizes the risks associated with direct cervical manipulation in C1-2 segment, in the presence of cervical vascular disorder [22]. Moreover, apart from the biomechanical effects (increase ROM, decrease passive and active stiffness), joint mobilization and manipulation have shown neurophysiological effects as changes in spinal excitability and in motor function, decrease in cortical excitability and activation in brain pain processing areas, increase activation in inhibitory pathways and changes in resting state brain functional connectivity [28].

Finally, cervical exercise is effective in reducing pain [29] and increasing cervical ROM in patients with neck pain [30]. Specifically, the low-load exercise on deep cervical flexor muscles has demonstrated effectiveness in improving its muscular parameters compared to other cervical exercise programs [31] and neuromuscular coordination [32]. This specific training is relevant as approximately 70% of patients with chronic neck pain exhibit decline in strength and endurance of these muscles [33]. Furthermore, apart from the neurophysiological effects, exercise may reduce kinesiophobia and fear of movement [34]. However, the immediate effect of exercise without associating manual therapy is not yet fully described.

Our hypothesis was that a one-session treatment of segments C0-1 and C2-3 can immediately improve the flexion-rotation test. The aim was to compare the effect, in the flexion-rotation test, of a 20-min session of manual therapy in C0-1 and C2-3 segment accompanied by cervical exercise, with the effect of a 20-min session of cervical exercise solely, in patients with chronic neck pain and positive flexion-rotation test.

## 2. Materials and Methods

### 2.1. Study Design

A prospective, longitudinal, randomized (simple 1:1), and controlled assessor-blind clinical trial was conducted. A researcher not involved in the study randomized the intervention for each patient using computer software (www.random.org). The randomization results were placed in a concealed opaque envelope, and participants were assigned to intervention groups.

The study was carried out in the facilities of the University of Zaragoza, Spain (Clinicaltrials.gov number: NCT04406753). This study complied with the ethical principles for research on human beings as per the Declaration of Helsinki (Fortaleza, Brazil, October 2013). It was approved by the local ethics committee (Comité Ético de Investigación Clínica en Aragón “CEICA”) in its Records n°13/2018 on 4 July 2018. All participants were required to provide written and informed consent to participate. This study followed all CONSORT criteria.

### 2.2. Sample Size Calculation

The primary variable used for sample size calculation was the flexion-rotation test ROM [2], obtaining a total sample of 48 subjects (24 subjects per group). The common standard deviation (7.15°) and the minimum difference to be detected between the groups (5.9°) were determined using the outcomes of Dunning et al. [2]. The sample size was calculated using the GRANMO 7.12 program, with an α risk of 0.05, test two-side, and a β risk of 0.20. 

### 2.3. Subjects

Forty-eight primary care subjects (12 men and 36 women) were included in the study. The inclusion criteria comprised: medical diagnosis of chronic neck pain with more than three months of evolution [35], a positive result in the flexion-rotation test (less than 33° or a difference of 10° or more between the two rotations) [6,8,9] and loss of mobility in C0-1 and/or C2-3 spine segments found through manual assessment according to Zito et al. and Kaltenborn [36,37]. Subjects were also required to be over 18 years old and sign the informed consent. The exclusion criteria were: contraindications for manual therapy or exercise, presenting warning signs or having suffered a relevant neck trauma, having participated in exercise or manual therapy programs in the last three months [22], an inability to maintain supine position, the use of pacemakers, an inability to perform the flexion-rotation test, language difficulties and pending litigation or lawsuits [38]. 

### 2.4. Measurements

The primary outcome measure in this study was the ROM in the flexion-rotation test. Secondary outcomes measures were neck pain intensity, pain intensity during the flexion-rotation test, and cervical ROM. 

The methodology proposed by Hall et al. [9] was followed to measure the flexion-rotation test. In order to perform it, the subject was placed in supine position, and the evaluator passively moved the patient’s cervical spine to its maximum flexion. Then the head was rotated to the right and left sides with the occiput resting against the evaluator’s abdomen. The movement stopped when either the subject presented symptoms, or the evaluator found a firm end feel, whichever situation occurred first [1,9]. If any pain appeared during the test, it was recorded by a verbal numeric pain rating scale (NPRS) (scale: 0 = no pain/ 10 = worst pain) for each rotation [39]. A Cervical Range of Motion (CROM) device (floating compass; Plastimo Airguide, Inc, Buffalo Groove, IL, USA) was used, and three measurements were taken for each rotation. The result was the mean of the three measurements [38]. The reliability of this test is excellent, ICC = 0.93–0.96 [4,40].

For current neck pain intensity at the time of evaluation, the NPRS was used (scale: 0 = no pain /10 = worst pain) [41]. NPRS has shown high reliability, with ICC = 0.76, in patients with chronic neck pain [42].

Active cervical ROM was measured in all cardinal planes for the assessment of cervical mobility. For active mobility testing, patients were asked to sit straight ahead, and to move their head as far as they could without pain in the different cardinal planes of movement [43]. A CROM device (floating compass; Plastimo Airguide, Inc.) was used and three measurements were taken for each movement. The result was the mean of the three measurements [38]. These measurements have high reliability (ICC = 0.98) [44].

A researcher specialized in manual therapy, with more than 5 years’ experience in physical therapy, performed the measures before and after the intervention. This researcher was blinded to the allocation group of each patient throughout the process. 

### 2.5. Intervention

Intervention was carried out by a different researcher with more than 5 years’ experience in physical therapy. The intervention was provided individually in the facilities at the University of Zaragoza. Participants in both groups received a single 20-min session.

### 2.6. Exercise Group

**Figure 1:**
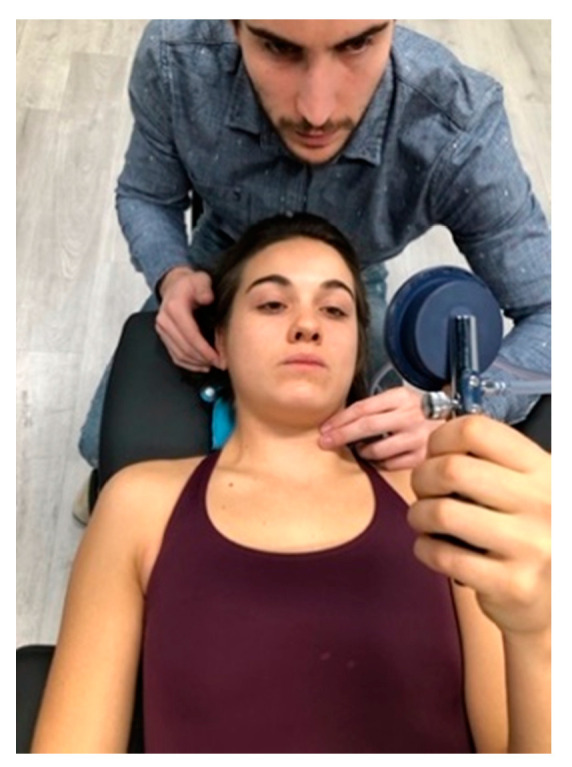
Deep neck flexor. Subjects were instructed to “gently nod their head as though they were saying ‘yes’”. Contribution from the superficial muscles was monitored using observation and palpation. Then, the subjects were taught to perform a slow and controlled deep neck flexor exercise.

After baseline assessments, patients began the cervical exercise. They were taught to perform the contraction of deep neck flexor muscles (longus capitis and longus colli) using the Stabilizer Pressure Biofeedback Unit (Chattanooga, TN, USA) in supine [45,46] (Figure 1). The exercise was always carried out without pain because pain can be an inhibitor of muscle contraction [47]. This exercise was chosen because it has been observed that patients with chronic neck pain show reduced activation of the deep cervical flexor muscles, which are considered important for control of stability of the spinal elements, which cannot be replicated by the more superficial anterior muscles training can successfully address impaired [14].

It is assumed that this altered recruitment pattern is a contributing factor to the chronification of neck pain. Training deep neck flexor muscles achieves the improvement of neuromuscular coordination and not of the strength and endurance, so a single session can obtain changes in the motor pattern [32].

The Exercise group carried out one 20-min session, composed of 2 blocks of 10 repetitions. They held each repetition for 10 s [47], and had a 40-s rest between each repetition and 2 min between blocks.

### 2.7. Manual Therapy + Exercise Group

**Figure 2:**
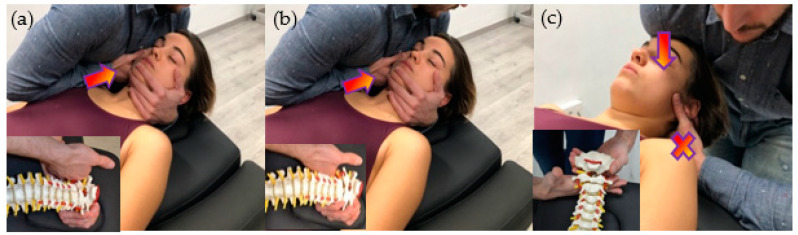
Manual therapy interventions. (**a**) C0-1 Traction Manipulation in the Resting Position. Patient was placed in supine with neck in a neutral position. The therapist gently cupped the patient’s chin with their hand while their arm was cradled around the head. The other hand placed the radial side of the index finger under the mastoid process and aligned the forearm in the line of drive pointing cranially. Then, the therapist applied a cranial thrust; (**b**) C2-3 Traction Manipulation in the Resting Position. The same handling procedure was performed, but the grip was relocated in C3 vertebrae; (**c**) C0-1 Dorsal Mobilization. Patient was positioned in supine, with neck in a neutral position. The therapist placed a hand dorsally at the level of the vertebral arch of C1 with the metacarpophalangeal and radial border of the index finger. The other hand was placed posteriorly under the occiput, with the shoulder positioned anteriorly on the patient’s forehead. The mobilization force was directed dorsally from the shoulder until the therapist felt a marked resistance, and then slightly more pressure was applied to perform a stretching mobilization.

The Manual Therapy+Exercise group (MT + E group) carried out a 20-min session of treatment. The manual therapy techniques were applied depending on the clinical findings in each patient, and the objective was to restore the mobility of C0-1 and C2-3 segments before applying cervical exercises. Manipulation (high-velocity low amplitude) and/or mobilization (low-velocity high amplitude) techniques of C0-1 and C2-3 segments were applied [1,3,48,49] (Figure 2). Manipulations were in the direction of traction, with the head in a neutral position [50]. A maximum of two trials at each level on each side was performed, yielding 2–8 thrusts [51]. Mobilization was performed repeating cycles of 45 s of mobilization and 15 s of rest [1,3]. The execution of the techniques is detailed in Figure 2. The cervical exercise performed by this group followed the same dose as the Exercise group, the only difference was that instead of performing 40-s rest between each exercise repetition, this group took 30-s. This was done in order to have 3 min of time to apply the manual therapy techniques before the exercise.

All the manipulation and mobilization techniques used in this trial follow IFOMPT recommendations to reduce the risk of adverse events [22].

### 2.8. Statistical Analysis

**Figure 3:**
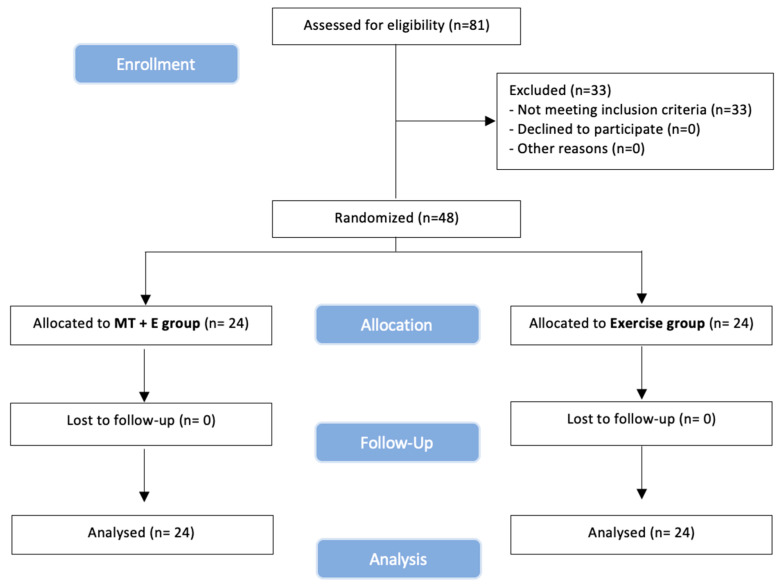
**CONSORT**. (Consolidated Standards of Reporting Trial) flow diagram.

Statistical analysis was conducted with the SPSS 23.0 package (IBM, Armonk, NY, USA). There was no loss of follow-up in the study. The mean, confidence interval and standard deviation were calculated for each variable. The Kolmogorov–Smirnov test was used to determine a normal distribution of quantitative data (*p* > 0.05). Outliers were examined. No value was excluded because extreme values did not cause any significant bias. Within- and between-group differences were analyzed using repeated measures of ANOVA and one-way ANOVA. If the assumption of sphericity was violated, the Greenhouse-Geisser correction was utilized for interpretation [51]. Effect sizes were calculated using Cohen’s d coefficient [52]. An effect size >0.8 was considered large; around 0.5, intermediate; and <0.2, small [52]. Exclusions after randomization are explained in Figure 3. All subjects enrolled originally were included in the final analysis as planned. Thus, participants were analyzed as per protocol (by intention-to-treat). The level of significance was set at *p* < 0.05.

The demographic characteristics of the sample are summarized in Table 1. No adverse events or side-effects were reported in any participant.

## 3. Results

Between June 2020 and July 2020, 81 volunteers were recruited. Forty-eight participants (12 men and 36 women) with a mean age of 51.9 (14.5) years old met all eligibility criteria and agreed to participate. Then, 24 participants were randomly assigned to each group, received their assigned treatment, and were analyzed for intention-to-treat (Figure 3). Enrollment and exclusions after randomization are in the flow diagram, see Figure 3.

### 3.1. Flexion-Rotation Test ROM and NPRS

No statistically significant changes were found in the within-group analysis (Table 2) for the exercise group. In the MT + E group, significant increase was found in the flexion-rotation test in right rotation (*p* < 0.001; d = 1.14) and left rotation (*p* < 0.001; d = 1.09) ROM. A statistically significant decrease in NPRS was also observed during both rotations (right NPRS rotation (*p* < 0.001; d = 0.89); left NPRS rotation (*p* < 0.001; d = 0.76)).

In the between-group analysis (Table 2), statistically significant differences were found in favor of the MT + E group in right rotation (*p* < 0.001; d = 1.72) and left rotation (*p* < 0.001; d = 1.65) ROM, and in right NPRS rotation (*p* < 0.001; d = 1.74) and in left NPRS rotation (*p* < 0.001; d = 1.41).

### 3.2. Neck Pain Intensity (Numeric Pain Rating Scale)

In the within-group analysis (Table 2) for the exercise group, no statistically significant changes were found at any time during the study. In the MT + E group, significant improvement was found (*p* < 0.001; d = 1.41).

In the between-group analysis (Table 2), statistically significant differences were found in favour of the MT + E group (*p* < 0.001; d = 1.30).

### 3.3. Cervical ROM

In the within-group analysis (Table 3) for exercise group, a statistically significant decrease was found in flexion (*p* < 0.008; d = 0.37), extension (*p* < 0.012; d = 0.26), and left side-bending (*p* < 0.030; d = 0.20). In the MT + E group, a significant increase was found only in left side-bending (*p* < 0.001; d = 0.43).

In the between-group analysis (Table 3), statistically significant differences were found in favor of the MT + E group in flexion (*p* < 0.038; d = 0.62), extension (*p* < 0.010; d = 0.77), right side-bending (*p* < 0.035; d = 0.63), left side-bending (*p* < 0.002; d = 0.93), right rotation (*p* < 0.006; d = 0.84), and left rotation (*p* < 0.001; d = 1.03).

## 4. Discussion

This study found an immediate increase in ROM on the flexion-rotation test in the MT + E group. The improvement in the flexion-rotation test observed in the MT + E group was superior to the minimal detectable change (4.7° for right rotation and 7.0° for left rotation) described by Hall et al. [40]. This group managed to overcome the clinically relevant improvement (10° or more) for the flexion-rotation test promoted by other authors [8,9]. In contrast, the exercise group did not experience any statistically significant change in this variable.

Except for the C1-2 segment, there is little information regarding the effects of the treatment of other cervical segments on upper cervical rotation ROM examined with the flexion-rotation test [1,3].

There is evidence that neck pain, ROM, and disability may be improved by treatment of segments and spinal regions remote from the symptomatic segment [24]. Hidalgo-García et al. [3] and Malo-Urriés et al. [1] found an increase in the flexion-rotation test when applying a mobilization in C0-1 segment. Carrasco-Uribarren et al. [50] evaluated the flexion-rotation test, and applied a protocol of traction manipulation in the resting position in C0-1, C1-2 and C2-3 segments, obtaining similar results to those observed in this study.

A possible explanation for these results in the MT + E group in our study may be due to the relationship between the articular, muscular, and ligamentous tissue between C0-1, C1-2 and C2-3 segments [3], particularly, by alar ligaments. C1-2 rotation is limited by the alar ligament system and occipital, and C2 are the main bony attachments of alar ligaments. Some authors have proposed that C0-1 [1,3] and/or C2-3 [50] dysfunction may inhibit the normal rotational mobility of C1-2 [53]. The MT + E group received a tailored manual therapy treatment according to the C0-1 and C2-3 dysfunction of each patient. This could be a potential explanation for the results of this study. Also, other non-biomechanical mechanisms such as spinal cord and central nervous system mechanisms may account for this improvement [3].

In our study, we found a significant reduction in NPRS for both rotations during the flexion-rotation test in the MT + E group. There is only one previous study that records NPRS during the flexion-rotation test [39], but the design of this study and the selection of the sample do not allow us to contrast our results.

A significant decrease in NPRS was found in MT + E group. In contrast, the exercise group did not experience any statistically significant change in neck pain intensity. Several studies found immediate improvements with manual therapy and exercise [54,55], with isolated manual therapy [56] or with isolated exercise [55,57].

Bialosky JE et al. [58] exposes the hypoalgesic effect of manual therapy and exercise through a model that is based on a mechanical stimulus initiating a chain of spinal neurophysiological events, peripheral events, and/or supraspinal events that would produce this hypoalgesia. Nevertheless, these neurophysiological effects would depend on the stimulus and the dose [58,59]. The upper cervical region is characterized by having a great number of mechanoreceptors, especially in the suboccipital region [60]. Therefore, this region has a high capacity to transmit mechanical inputs from manual techniques, resulting in a decrease in neck pain intensity in MT + E group. There is a non-significant increase in NPRS in the exercise group. This may be due to tissue irritation processes because of exercising deep cervical muscles through upper cervical spine flexion without a proper ROM. There are studies that suggested that irritation of the suboccipital tissues can cause an increase in muscle stretch reflex, including in adjacent muscles, generating an increase in tissue sensitivity [61,62,63]. Because these patients had a rotatory restriction in the upper cervical spine, the irritation could affect central processes produced by activation of segmental inhibitory pathways, spinal cord pathways, or descending inhibitory pathways from the brainstem generating an increase in pain [64,65]. Although deep cervical flexor exercises are an evidence-based treatment in patients with chronic cervical pain, the Exercise group did not perform a specific exercise program for the upper cervical dysfunction and this could also explain the results of the exercise group in our sample.

A significant increase in the left side-bending was found in the MT + E group. In contrast, we found a significant decrease in the cervical ROM in the exercise group in flexion, extension, and left side-bending movements.

The measuring inaccuracy in the side-bending of the CROM range is between 5° and 10° [66]. In this study, the magnitude of observed change in both groups was inferior to 5°. Therefore, we cannot confirm that the increases or the decreases in left side-bending are due to the interventions. However, several studies have conducted interventions based on manual therapy with or without exercise on deep flexor muscles. These yield improvements or no changes in cervical ROM, yet none yield decreases [55,67]. Our findings may differ because this study is the first to carry out these types of interventions in patients with chronic neck pain with dysfunction of the upper cervical spine.

One study explains that a full ROM in the upper cervical spine is needed to be able to properly contract the deep flexor muscles [63]. It is possible that the ROM deficit in the upper cervical spine made it difficult to carry out the prescribed deep flexor muscles exercise. This possibly favors an excess of muscular recruitment to try to overcome upper cervical spine restriction, and that translates into a decrease in cervical ROM in the exercise group [48]. Two studies documented that activation of cervical muscles, either for motion production or providing stability, would inevitably increase the loading of the spine [68,69]. For this reason, the joint compression that occurs with muscle activation when there is an upper cervical spine dysfunction would be higher than in physiological conditions [48]. Further studies would be needed to verify this possibility.

Finally, the results of this study support the hypothesis that mobilization and manipulation of C0-1 and C2-3 is an effective treatment for restoring upper cervical spine rotation measured by the flexion-rotation test. These results are consistent with other studies examining the effect of mobilization and manipulation treatments targeting the C1-2 segment [2,18]. However, in contrast to these studies, our study did not position the upper cervical in the end range of rotation. This technique follows IFOMPTs recommendation promotion of applying cervical mobilization or manipulation near to the neutral neck position [22].

### Limitations

The main limitation of this study was that only the immediate effects of the techniques were measured. Future studies with short-, mid-, and long-term follow-ups in this group of patients with a positive flexion-rotation test using these treatment techniques within a multimodal approach (including specific cervical and thoracic manual therapy, exercise and pain education) would be necessary.

An important limitation was the possible representativeness of this subgroup of chronic neck pain patients with positive flexion-rotation test, and the external validity among all the possible subgroups of patients suffering from this syndrome.

Another significant limitation was that we do not know what specific intervention has had the most significant impact on the patients. Different studies have shown good effects treating the C0-1 segment [1,3], but we do not know if the isolated treatment of C2-3 can improve the flexion-rotation test.

Finally, the measuring inaccuracy of the CROM range is between 5° and 10° [66]. Therefore, we cannot assure that the significant worsening of the exercise group ROM is due to the intervention received, as it is possible that it is due to the CROM device.

It would be necessary to undertake more studies with this subgroup of patients with chronic neck pain and positive flexion-rotation test to understand their behavior.

## 5. Conclusions

This study showed that one 20-min manual therapy session of C0-1 and C2-3 segments with exercise can immediately improve flexion-rotation test and pain intensity in patients with chronic neck pain and positive flexion-rotation test.

## Figures and Tables

**Table 1:** Baseline Features for Both Groups.

	E Group (*n* = 24)	MT + E Group (*n* = 24)
Clinical features		
Age (years)	54.71 ± 14.59	49.17 ± 14.24
Sex (female)	19 (79.2%)	17 (70.8%)
Duration of Symptoms (months)	141.67 ± 157.27	91.88 ± 85.51
NPRS (0–10)	4.34 ± 2.65	4.13 ± 1.70
Flexion-rotation test		
Right (°)	16.93 ± 10.17	22.11 ± 10.35
Left (°)	18.97 ± 10.26	24.06 ± 9.33
Right (NPRS)	5.08 ± 2.32	3.67 ± 2.35
Left (NPRS)	4.75 ± 2.67	2.34 ± 2.37
Cervical ROM (°)		
Flexion	47.08 ± 10.77	47.38 ± 11.50
Extension	50.71 ± 13.44	54.54 ± 14.56
Right Side-Bending	26.92 ± 8.91	32.29 ± 9.95
Left Side-Bending	28.00 ± 9.17	30.13 ± 9.63
Right Rotation	52.29 ± 12.48	57.34 ± 16.02
Left Rotation	54.58 ± 12.40	60.25 ± 15.61

Abbreviations: NPRS. Numerical Pain Rating Scale; ROM. Range of motion; E. Exercise; MT + E. Manual Therapy+Exercise.

**Table 2:** Pre- and post-treatment in NPRS and flexion-rotation test outcomes.

Outcome	Group	Pre-Treatment	Post-Treatment	Within-Group ^†^	Between-Group ^‡^
NPRS (0–10)	E Group	4.34 ± 2.65 (3.2, 5.5)	4.50 ± 2.49 (3.5, 5.6)	*p* > 0.709	*p* < 0.001 d = 1.30
				d = 0.06	
	MT + E Group	4.13 ± 1.70 (3.4, 4.8)	1.75 ± 1.68 (1.0, 2.5)	*p* < 0.001	
				d = 1.41	
Flexion-rotation test (R) (°)	E Group	16.93 ± 10.17 (12.6, 21.2)	16.21 ± 10.56 (11.8, 20.7)	*p* > 0.090	*p* < 0.001 d = 1.72
				d = 0.07	
	MT + E Group	22.11 ± 10.35 (17.7, 26.5)	33.15 ± 9.06 (29.3, 37.0)	*p* < 0.001	
				d = 1.14	
Flexion-rotation test (L) (°)	E Group	18.97 ± 10.26 (14.6, 23.3)	17.98 ± 10.43 (13.1, 22.2)	*p* >0.221	*p* < 0.001 d = 1.65
				d = 0.10	
	MT + E Group	24.06 ± 9.33 (20.3, 28.2)	34.17 ± 9.16 (30.2, 37.9)	*p* < 0.001	
				d = 1.09	
Flexion-rotation test (R) (NPRS 0–10)	E Group	5.08 ± 2.32 (4.1, 6.1)	5.50 ± 2.27 (4.5, 6.5)	*p* > 0.135	*p* <0.001 d = 1.74
				d = 0.18	
	MT + E Group	3.67 ± 2.35 (2.7, 4.7)	1.67 ± 2.12 (0.8, 2.6)	*p* < 0.001	
				d = 0.89	
Flexion-rotation test (L) (NPRS 0–10)	E Group	4.75 ± 2.67 (3.6, 5.9)	4.92 ± 2.54 (3.9, 6.0)	*p* > 0.548	*p* < 0.001 d = 1.41
				d = 0.07	
	MT + E Group	3.34 ± 2.37 (2.3, 4.3)	1.67 ± 2.04 (0.8, 2.5)	*p* < 0.001	
				d = 0.76	

Abbreviations: R. right; L. left; NPRS. Numerical Pain Rating Scale E. Exercise; MT + E. Manual Therapy+Exercise. ^†^**,** value is repeated measures ANOVA. ^‡^**,** value is one-way ANOVA. *p* values ≤ 0.05 are statistically significant. d. Cohen d effect size.

**Table 3:** Pre- and post-treatment in cervical range of motion outcomes.

Outcome/Group	Pre-Treatment	Post-Treatment	Within-Group ^†^	Between-Group ^‡^
Flexion (°)				
E Group	47.08 ± 10.77 (42.5, 51.6)	43.67 ± 10.82 (39.1, 48.2)	*p* < 0.008	*p* < 0.038 d = 0.62
			d = 0.37	
MT + E Group	47.38 ± 11.50 (42.5, 52.2)	49.83 ± 9.13 (46.0, 53.7)	*p* > 0.148	
			d = 0.24	
Extension (°)				
E Group	50.71 ± 13.44 (45.0, 56.4)	47.46 ± 11.31 (42.7, 52.2)	*p* < 0.012	*p* < 0.010 d = 0.77
			d = 0.26	
MT + E Group	54.54 ± 14.56 (48.4, 60.7)	56.79 ± 12.81 (51.4, 62.2)	*p* > 0.171	
			d = 0.16	
Side-bending (Right) (°)				
E Group	26.92 ± 8.91 (23.2, 30.7)	26.54 ± 8.46 (23.0, 30.1)	*p* > 0.531	*p* < 0.035 d = 0.63
			d = 0.04	
MT + E Group	32.29 ± 9.95 (28.1, 36.5)	32.04 ± 9.04 (28.2, 35.9)	*p* > 0.846	
			d = 0.03	
Side-bending (Left) (°)				
E Group	28.00 ± 9.17 (24.1, 31.9)	26.25 ± 8.68 (22.6, 29.9)	*p* < 0.030	*p* < 0.002 d = 0.93
			d = 0.20	
MT + E Group	30.13 ± 9.63 (26.1, 34.2)	33.79 ± 7.47 (30.6, 36.9)	*p* < 0.001	
			d = 0.43	
Rotation (Right) (°)				
E Group	52.29 ± 12.48 (47.0, 57.6)	50.58 ± 12.45 (45.3, 55.8)	*p* > 0.063	*p* < 0.006 d = 0.84
			d = 0.14	
MT + E Group	57.34 ±16.02 (50.6, 64.1)	61.13 ± 12.75 (55.7, 66.5)	*p* > 0.168	
			d = 0.26	
Rotation (Left) (°)				
E Group	54.58 ± 12.40 (49.4, 59.8)	52.67 ± 12.26 (47.5, 57.8)	*p* > 0.163	*p* < 0.001 d = 1.03
			d = 0.16	
MT + E Group	60.25 ± 15.61 (53.7, 66.8)	64.75 ± 11.15 (60.0, 69.5)	*p* > 0.082	
			d = 0.33	

Abbreviations: E. Exercise; MT + E. Manual Therapy+Exercise. ^†^**,** value is repeated measures ANOVA. ^‡^**,** value is one-way ANOVA. *p* values ≤ 0.05 are statistically significant. d. Cohen d effect size.

## Data Availability

You can find all the data from this study in the “HARVARD Dataverse” using the following link: https://dataverse.harvard.edu/dataset.xhtml?persistentId=doi:10.7910/DVN/XYOQDR&version=DRAFT.
